# Enzymatic Degradation of *Gracilariopsis lemaneiformis* Polysaccharide and the Antioxidant Activity of Its Degradation Products

**DOI:** 10.3390/md19050270

**Published:** 2021-05-12

**Authors:** Tian Fang, Xiaoqian Zhang, Shanshan Hu, Yanyan Yu, Xue Sun, Nianjun Xu

**Affiliations:** Key Laboratory of Marine Biotechnology of Zhejiang Province, School of Marine Sciences, Ningbo University, Ningbo 315211, China; fangtian1027@163.com (T.F.); Hushanshanznb@163.com (S.H.); Yuyanyannbu@163.com (Y.Y.); sunxue@nbu.edu.cn (X.S.)

**Keywords:** *Gracilariopsis lemaneiformis*, polysaccharides, enzymatic degradation, antioxidant activity, oxidative damage

## Abstract

*Gracilariopsis lemaneiformis* polysaccharides (GLP) were degraded using pectinase, glucoamylase, cellulase, xylanase, and β-dextranase into low-molecular-weight polysaccharides, namely, GPP, GGP, GCP, GXP, and GDP, respectively, and their antioxidant capacities were investigated. The degraded GLP showed higher antioxidant activities than natural GLP, and GDP exhibited the highest antioxidant activity. After the optimization of degradation conditions through single-factor and orthogonal optimization experiments, four polysaccharide fractions (GDP1, GDP2, GDP3, and GDP4) with high antioxidant abilities (hydroxyl radical scavenging activity, DPPH radical scavenging activity, reduction capacity, and total antioxidant capacity) were obtained. Their cytoprotective activities against H_2_O_2_-induced oxidative damage in human fetal lung fibroblast 1 (HFL1) cells were examined. Results suggested that GDP pretreatment can significantly improve cell viability, reduce reactive oxygen species and malonaldehyde levels, improve antioxidant enzyme activity and mitochondria membrane potential, and alleviate oxidative damage in HFL1 cells. Thus, the enzyme degradation of GLP with β-dextranase can significantly improve its antioxidant activity, and GDP might be a suitable source of natural antioxidants.

## 1. Introduction

Oxidative stress is caused by the excessive levels of reactive oxygen species (ROS), resulting in the imbalance of redox state in cells [1]. Oxidative stress is involved in cardiovascular diseases and other pathophysiological processes, such as diabetes, cancer, and tissue injury [2,3]. In the last decades, natural antioxidants have gained attention, owing to their promising bioactivities and fewer side effects. Among the natural products, mounting seaweed-derived polysaccharides have been identified and proved to exhibit excellent antioxidant activities by scavenging ROS and alleviating oxidative damage in organisms [4]. Polysaccharides from *Gracilaria rubra* showed strong DPPH scavenging ability (22.84–41.59%) at 2.5 mg/mL [5]. Lee et al. (2016) [6] also prepared antioxidant polysaccharides from *Pyropia yezoensis* with a microwave-assistant rapid enzyme digest system.

As a potential valuable resource for food and medicine additive, marine red alga *Gracilariopsis lemaneiformis* is rich in a variety of bioactive substances, such as carbohydrates, proteins, dietary fibers, and minerals, and *G. lemaneiformis* polysaccharides (GLP) are the most important functional components. Numerous pieces of evidence have confirmed that GLP possess various biological activities, such as immunomodulation, antitumor, antiradiation, prebiotic activities, and antioxidant effects [7,8]. GLP, a linear polysaccharide with −OSO_3_H groups, are composed of alternating (1 → 3)-linked D-galactose and (1 → 4)-linked 3,6-anhydro-L-galactose units [9]. Notably, the physiochemical properties of GLP might vary with the difference of its sources, extraction, and purification method [10]. Our previous research prepared GLP by the traditional method of hot-water extraction and obtained a polysaccharide with Mw of 123.06 kDa and sulfate content of 9.24% [7]. Studies on in vitro antioxidant activity of GLP indicated that the GLP had strong hydroxyl radical scavenging activity [11] and could protect human kidney proximal tubular epithelial cells (HK-2) from oxidative stress-mediated damage [12]. However, the biological activities of polysaccharides are closely associated with their primary structures, such as monosaccharide composition, sulfate and uronic acid contents, and molecular weight (Mw) [13,14]. The bioavailability and bioactivity of polysaccharides with high Mw can be restricted because of their high viscosity, poor solubility, and difficulty in penetrating the cell membrane [15]. The bioactivity of natural polysaccharides can be improved through degradation, which can alter the physicochemical properties of a polysaccharide, such as molecular weight, sulfate content, and solubility [16,17]. The repair abilities of degraded low-molecular-weight seaweed polysaccharides were stronger than that of the undegraded polysaccharide on oxalate-induced damaged HK-2 cells, and a positive correlation was found between -SO_3_H content and repair ability [18]. Similarly, ultrasonic degradation decreased the molecular weight of *P. yezoensis* polysaccharides and enhanced its superoxide and hydroxyl radical scavenging abilities [19].

Degradation methods influence the structures and activities of polysaccharides. Physical, chemical, and biological methods have been widely used in polysaccharides degradation. Biodegradation methods, particularly enzymatic degradation, can specifically break glycoside bonds and yield homogeneous products in a mild manner. According to Hélène Barreteau et al. (2006) [20], the enzymatic degradation of polysaccharides is the best option for large-scale oligosaccharide production and oligosaccharides with desired molecular weights may be obtained through enzymatic hydrolysis. Effects of hydrochloric acid, ultrasonic-assisted hydrochloric acid, and α-amylase degradation on *Passiflora edulis* Sims peel polysaccharides were investigated, and the results showed that enzymatic degradation could yield products with strong antioxidant activities and cause minimal damage to the triple helix structures of polysaccharides [21]. Previous studies have degraded GLP by chemical method with H_2_O_2_, citric acid, or vitamin C [12,22]. However, to our knowledge, the effect of enzyme degradation on the antioxidant activities of GLP is scarcely addressed.

The aim of this study was to improve the physicochemical properties of GLP and obtain polysaccharides with better antioxidant activity. Five carbohydrases, namely, pectinase, glucoamylase, cellulase, xylanase, and β-dextranase, were used for the first time to degrade GLP, and degradation conditions (time, temperature, pH, and enzyme dosage) were optimized through the in vitro chemical assays of antioxidant activities (hydroxyl radical scavenging activity, 1,1-diphenyl-2-trinitrophenyl hydrazine (DPPH) radical scavenging activity, reduction capacity, and total antioxidant capacity) of enzymatic hydrolysates. Moreover, human fetal lung fibroblast 1 (HFL1) cells are ideal models for studying antioxidant activity [23]. Thus, the antioxidant activities of three degraded polysaccharides obtained under optimal conditions were evaluated with HFL1 cells at the cellular level.

## 2. Results and Discussion

### 2.1. Chemical Analysis of Polysaccharides

The chemical compositions of the GLP and hydrolysis products produced by pectinase (GPP), glucoamylase (GGP), cellulose (GCP), xylanase (GXP), and β-dextranase (GDP) were obtained and shown in Table 1. The total sugar contents of the degradation products, except GXP, significantly increased relative to that of GLP, and GDP and GGP had the highest contents (Table 1). The contents of sulfate and protein were 18.71% and 1.10% in GLP, respectively, indicating that GLP was a sulfated polysaccharide. Moreover, enzymolysis had significant effects on the sulfate and uronic acid levels of the degradation products of the GLP. The sulfate contents of the degradation products except GGP decreased, and GCP possessed the lowest sulfate content (Table 1). Most of the GLP hydrolysates were desulfated at varying degrees, possibly because of long periods of degradation in an acidic environment [24]. After degradation, the contents of uronic acid in GPP and GXP obviously increased, whereas the content in GCP significantly decreased. The molecular weights of the GLP hydrolysates decreased at varying degrees (Table 1). Among them, GPP had the highest decrease in Mw, which dropped from 107 to 66 kDa, followed by the Mw of GDP, which decreased to 81 kDa.

Monosaccharide composition will affect the activity of polysaccharides. As shown in Table 2, GLP was mainly composed of Gal (89.7%) with a lower level of Glu (2.3%) and Xyl (1.2%), which was consistent with the results obtained by Shi et al. (2018) [25]. Enzymatic degradation significantly changed the monosaccharide composition of GLP. The degradation products showed a much higher Glu level than GLP and mainly consisted of Gal and Glu. The molar ratios of Gal and Glu in GLP, GPP, GGP, GCP, GXP, and GDP were 39.0:1, 1.8:1, 4.9:1, 0.3:1, 3.0:1, and 1.0:1, separately. In addition, GPP, GGP, and GDP contained a certain amount of Rha and Ara, while Man existed in GGP (10.9%), GCP (4.2%), GXP (0.8%), and GDP (11.1%). The differences in monosaccharide composition of these samples might be explained by the different hydrolyzed sites of different enzymes.

### 2.2. Fourier Transform Infrared Spectra (FT-IR) Analysis

FT-IR analysis was used in studying the structures of the polysaccharides (GLP and its enzymatic degradation products) according to the characteristic absorption feature. Figure S1 showed that GLP, GPP, GGP, GCP, GXP, and GDP had the typical absorptive peaks of glycosidic structures. The broad, strong absorptive peak at 3363.25–3403.74 cm^−1^ indicated that the presence of many intramolecular and intermolecular hydrogen bonds featuring hydroxyl stretching vibration. The absorption peak at approximately 2901.38–2936.09 cm^−1^ was a C–H stretching vibration peak, and the relatively strong absorption peak at 1594.84–1648.84 cm^−1^ was caused by the asymmetric and symmetric stretching vibrations of COOH [18]. All samples had absorption bands near 1373.07–1413.57 cm^−1^ due to the variable angle vibration of C–H. The characteristic absorption peaks at 1245.79–1255.43 cm^−1^ were attributed to the S=O vibration of the sulfate groups in GLP and its degradation products, which was consistent with the results in Table 1. The peaks at 1036.55–1075.12 cm^−1^ were assigned to glycosidic linkage C-O stretching vibration. The stretching vibration of C=O at 931.45–933.38 cm^−1^ indicated the existence of 3,6-endo-galactose in GLP and its degradation products. The peak at 845.63–894.81 cm^−1^ indicated the existence of a β-glycosidic bond [26]. Moreover, most of the absorption bands of GLP were retained in the degradation products of the GLP, indicating that the structures of the GLP were not destroyed after enzymatic degradation. However, the infrared spectra of the degradation products were slightly different from those of the GLP, and the stretching and variable angle vibration peaks of C–H moved to higher wave numbers, indicating that the intramolecular and intermolecular hydrogen bonds of the degradation products were enhanced.

### 2.3. Screening of Degradation Enzymes

**Figure 1:**
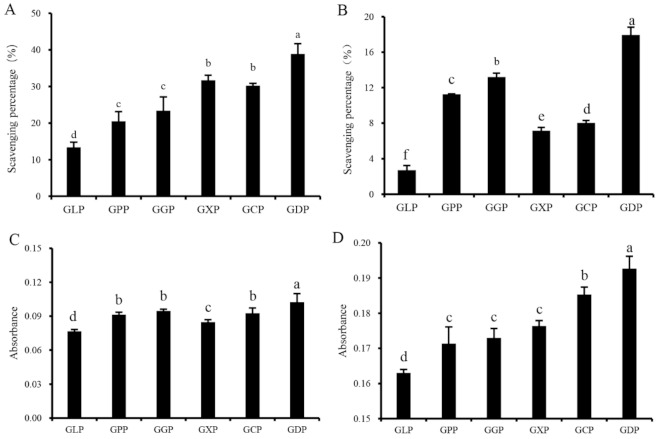
Antioxidant activities of GLP and different enzymatic hydrolysis products at a concentration of 1.0 mg/mL; scavenging ability of hydroxyl radical (**A**), scavenging ability of DPPH radical (**B**), reducing capacity (**C**), and total antioxidant capacity (**D**). Vertical bars represent means ± SD (n = 3), and different letters indicate significant difference (*p* < 0.05).

Natural antioxidants play an important role in inhibiting ROS formation and eliminating ROS in organisms. The scavenging abilities of hydroxyl radical and DPPH, reducing capacity, and total antioxidant capacity are widely used for evaluating the radical scavenging abilities of antioxidants in vitro [27,28]. Figure 1A–D suggested that the in vitro antioxidant activities of enzymatically degraded products (GPP, GGP, GXP, GCP, and GDP) were significantly higher than those of the GLP. At a concentration of 1.0 mg/mL, the scavenging rates of GLP on hydroxyl radical and DPPH radical were 13.39% and 2.70%, respectively (Figure 1A,B). However, GDP exhibited the most effective antioxidant activity against hydroxyl and DPPH radicals, with scavenging rates of 38.86% and 17.95%, respectively (Figure 1A,B). Consistently, GDP had higher reducing and total antioxidant capacities than the other degradation products (Figure 1C,D). Thus, β-dextranase was considered as an optimal enzyme for preparing the antioxidant polysaccharides of *G. lemaneiformis*. Polysaccharides with lower molecular weight exhibit higher antioxidant activity than those with high molecular weight [6]. Interestingly, the Mw of GDP was 81 kDa, which is lower than that of GLP (106 kDa) but higher than that of GPP (66 kDa) (Table 1), indicating that GLP with suitable molecular weight may exhibit better antioxidant ability [4,12]. In addition, monosaccharide composition also affects the antioxidant activity of polysaccharides. The antioxidant activities of *Lentinula edodes* polysaccharides rely on the ratios of different monosaccharides in the composition, and Ara and Man positively modulate the antioxidant properties of *L. edodes* polysaccharides [29]. In our present study, the Ara and Man contents in GDP and GGP significantly increased than other polysaccharides (Table 2), which was generally consistent with their increased antioxidant activities (Figure 1). It was inferred that the antioxidant effect of GLP was closely related to the Mw and monosaccharide composition of polysaccharides. Notably, it was reported that the sulfated derivative of GLP showed a stronger superoxide anion scavenging ability than GLP [30]. However, no correlation was observed between antioxidant activity and the sulfate contents of polysaccharides prepared in our present study (Table 1, Figure 1), which was consistent with the results obtained by Tang et al. (2017) [31]. These findings may be ascribed to the fact that the molecular weight and chemical composition were varied among GLP and its degradation products, and the antioxidant activities of polysaccharides are associated not only with a single factor but also with the combined actions of several factors, such as molecular weight, sulfate and uronic acid content, monosaccharide composition [14].

### 2.4. Single-Factor Experiment

The optimal hydrolysis conditions of β-dextranase were screened through single-factor and orthogonal optimization experiments. Enzyme activity is affected by hydrolysis time, temperature, and pH [11]. In the present study, the hydrolysis conditions of GLP, including time, temperature, pH, and enzyme dosage, were optimized, and the hydroxyl radical scavenging, DPPH radical scavenging, reducing, and total antioxidant abilities were measured for the assessment of the antioxidant activity of GDP obtained at different hydrolysis conditions. The antioxidant activities of GDP improved at its concentration increased from 0.2 to 1.0 mg/mL in the present study (Figures S2–S5).

The effects of enzymolysis time (60, 90, 120, and 150 min) on GLP degradation were studied at a hydrolysis temperature of 50 °C, pH of 5.0, and enzyme dosage of 300,000 U/g (Figure S2). At 0.2–1.0 mg/mL, the DPPH radical scavenging ability, reducing ability, and total antioxidant ability of the degradation products increased significantly at an enzymatic hydrolysis time of 90 min, whereas decreased from 90 to 150 min (Figure S2). These results may be explained by the complete degradation of GLP with the extension of time at the initial stage of degradation, and the excessive degradation of polysaccharides may have occurred after 90 min of degradation, leading to structural changes and decreased activity of the polysaccharides [32,33]. Therefore, the optimal hydrolysis time for the production of antioxidant polysaccharides from *G. lemaneiformis* was 90 min.

Temperature is an important factor affecting enzyme activity [11,34]. The effects of hydrolysis temperature on the antioxidant activity of GDP in vitro are displayed in Figure S3. Scavenging abilities against hydroxyl and DPPH radicals, reducing capacity, and total antioxidant capacity of GDP increased from 30 to 40 °C (Figure S3). A high temperature can accelerate the molecular movement and ensure full contact between a substrate and enzyme [35]. When the temperature exceeded 40 °C, the antioxidant activity of GDP decreased, possibly because of the degradation and inactivation of the enzyme at a high temperature [36]. Therefore, 40 °C was regarded as the best temperature for GLP degradation.

pH has a great influence on enzyme activity. Enzymes have catalytic activities only within a certain pH range [37]. As shown in Figure S4, the antioxidant activity of GDP improved with increasing pH, and the strongest antioxidant activity of GDP was observed at pH 5.0. It was inferred that pH exceeding 5.0 was beyond the suitable pH range of β-dextranase and led to decreases in enzyme activity and poor degradation effect. Thus, pH 5.0 was considered optimal in the present study.

The effects of enzyme dosages of 200,000–350,000 U/g on the antioxidant activity of GDP were studied. GLP was hydrolyzed by β-dextranase effectively, and enzyme dosage is vital for the antioxidant activity of polysaccharide hydrolysates (Figure S5). Antioxidant activity increased at enzyme dosages of 200,000–300,000 U/g (Figure S5) but decreased rapidly when the enzyme dosage exceeded 300,000 U/g, possibly because of the limited substrate concentration, which limited the interaction between substrates and enzymes or because the polysaccharide was wrapped in the enzyme [38].

### 2.5. Orthogonal Optimization Experiment and Verification Test

According to the results obtained from the single-factor experiment, the hydrolysis conditions of GLP, including temperature, pH, and enzyme dosage, were optimized using an orthogonal experimental design. According to the *R* values, the effects of different factors on hydroxyl radical scavenging capacity were in the descending order of enzyme dosage, pH, and temperature (Table 3), and the optimal degradation conditions (A_1_B_1_C_1_) were 30 °C, pH 4.4, and 250,000 U/g (Table 3, Table S1). Range analysis also showed that temperature exerted the highest effect on DPPH scavenging capacity (Table 3). The degradation products with the highest DPPH scavenging activities were obtained under the conditions of A_1_B_1_C_3_, in which the temperature, pH, and enzyme dosage were 30 °C, 4.4, and 350,000 U/g, respectively (Table 3, Table S1). Temperature and pH were the most important factors affecting the reduction capacity and total antioxidant capacity of GLP hydrolysates (Table 3). The optimal degradation conditions were as follows: 50 °C, pH 5.6, 350,000 U/g for reduction capacity (A_3_B_3_C_3_) and 40 °C, pH 5.0, 300,000 U/g for total antioxidant capacity (A_2_B_2_C_2_) (Table 3, Table S1). A comprehensive scheme (A_1_B_1_C_1_) was obtained through the comprehensive analysis of the results of the four antioxidant indexes (Table 3, Table S1). It seemed that the optimum degradation conditions were different when focusing on different evaluation indexes.

The verification experiments were further carried out with 1.0 mg/mL of GDPs, and the results were shown in Table 4. GDP3 showed the strongest hydroxyl radical scavenging ability (61.99%) (Table 4), which was higher than that of degradation products obtained by chemical degradation of GLP [39]. GDP2, prepared under the conditions of 30 °C, pH 4.4, and 350,000 U/g, exhibited the highest DPPH radical scavenging activity (32.68%; Table 4). In addition, GDP4 showed the strongest reduction ability and total antioxidant capacity in the four validation samples (Table 4). At 1.0 mg/mL, the reduction and total antioxidant capacities were 0.140 and 0.355, respectively, which were significantly enhanced compared with those of GLP. The antioxidant activities of GDP2, GDP3, and GDP4 in the HFL1 cells were further evaluated at the cellular level.

### 2.6. GDP Alleviated H_2_O_2_-Induced Oxidative Injury in HFL1 Cells

**Figure 2:**
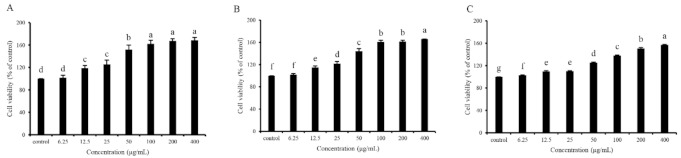
Effects of GDP2 (**A**), GDP3 (**B**), GDP4 (**C**) on the cell viability of HFL1 cells. Vertical bars represent means ± SD (n = 4), and different letters indicate significant difference (*p* < 0.05).

**Figure 3:**
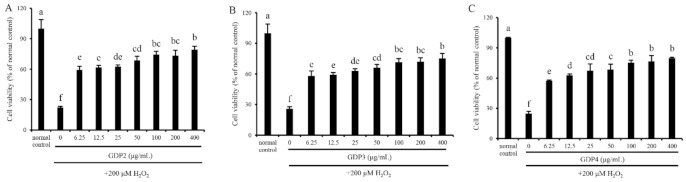
Protective effects of GDP2 (**A**), GDP3 (**B**), and GDP4 (**C**) against H_2_O_2_-induced injury in the HFL1 cells. Vertical bars represent means ± SD (n = 4), and different letters indicate significant difference (*p* < 0.05).

In vitro cell assays for screening antioxidants are vital since the mechanism of the antioxidants on human health benefits goes beyond their antioxidant ability, and their bioavailability, cytotoxicity, and other bioactivities also matter [40]. Cell viability determination through the MTT method was performed to evaluate the cytotoxicity and antioxidant capacity of GDP2, GDP3, and GDP4 on H_2_O_2_-induced oxidative damage in HFL1 cells. HFL1 cells treated with different concentrations (6.25–400 μg/mL) of GDPs (GDP2, GDP3, and GDP4) for 24 h significantly increased the cell viability compared with the control (Figure 2), showing that GDPs had no cytotoxicity to HFL1 cells. This finding was also observed in seaweed polysaccharides from *S. fusiforme*, which improved the cell viability of the HaCaT cells [41]. H_2_O_2_-induced oxidative stress has been widely used in investigating the antioxidant abilities of various bioactive substances [42]. The cell viability of HFL1 cells decreased to approximately one-fourth of that of normal cells after exposure to 200 μM H_2_O_2_ for 6 h, indicating that the establishment of the H_2_O_2_-induced oxidative damage model was successful (Figure 3). Moreover, Figure 3 suggests that pretreatment with GDPs can significantly improve the cell viability of oxidative damaged HFL1 cells. Compared with the negative control group (only H_2_O_2_ was added), the cell viability of GDP2, GDP3, and GDP4 treatment groups significantly increased by 167.48%, 125.06%, and 132.00% at a concentration of 6.25 μg/mL, respectively (Figure 3), indicating that GDPs possessed excellent cytoprotection effect on oxidative damaged HFL1 cells. Notably, GDP2 was the most effective, followed by GDP4 and GDP3, which was in accordance with their DPPH scavenging ability, and the best cytoprotection effect of GDP2 may be ascribed to its better DPPH free radical scavenging ability (Table 4). Moreover, the cell viability of the HFL1 cells increased gradually with GDPs concentration in a dose-dependent manner. The cell viability in GDP2, GDP3, and GDP4 groups increased 2.6, 1.9, and 2.2 times, respectively, at a concentration of 400 μg/mL, and no significant difference was among the 100, 200, and 400 mg/L treatments. Thus, 200 μg/mL of GDP was selected for the further investigation of the antioxidant activities of GDP on HFL1 cells.

### 2.7. GDP Reduced ROS Generation in the HFL1 Cells

**Figure 4:**
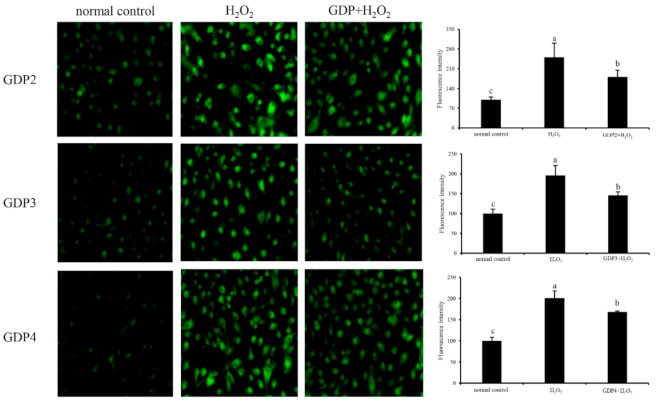
Effects of GDP2, GDP3, and GDP4 on ROS generation in the HFL1 cells. Vertical bars represent means ± SD (n = 4), and different letters indicate significant difference (*p* < 0.05).

ROS is a normal metabolite that plays an important role in cell signaling pathways [43]. However, excessive ROS can lead to oxidative damage in the human body and food system by reacting with biomolecules, such as DNA, lipids, proteins, and sugars, and destroying their structures and functions [44]. Intracellular ROS is an efficient index for oxidative stress evaluation in living cells [45]. Effects of GDPs on intracellular ROS generation in damaged HFL1 cells were investigated using a DCFH-DA fluorescence probe. The green fluorescence intensity of DCFH-DA was proportional to ROS level [46]. In the present study, the level of intracellular ROS in the HFL1 cells increased significantly after 6 h of H_2_O_2_ treatment (Figure 4), indicating that H_2_O_2_ treatment significantly induced the production of ROS in the HFL1 cells. However, after pretreatment with 200 μg/mL GDP2, GDP3, and GDP4, intracellular ROS levels in the HFL1 cells decreased by 69.42%, 50.37%, and 32.33% compared with that in the negative control cells, respectively, supporting that GDPs effectively scavenged or inhibited ROS production induced by H_2_O_2_ in HFL1 cells.

### 2.8. GDP Alleviated Lipid Peroxidation Level and Increased the Cellular Antioxidant Enzymes Activities of HFL1 Cells

**Figure 5:**
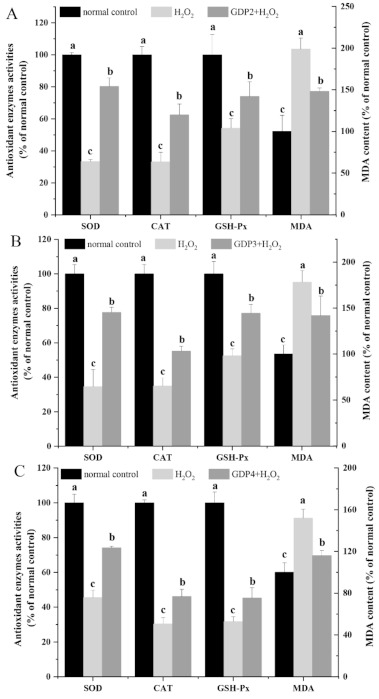
Effects of GDP2 (**A**), GDP3 (**B**), and GDP4 (**C**) on MDA content and SOD, CAT, and GSH-Px activities in the H_2_O_2_-damaged HFL1 cells. Vertical bars represent means ± SD (n = 4), and different letters indicate significant difference (*p* < 0.05).

Malondialdehyde (MDA), one of the most important products of the lipid peroxidation of unsaturated fatty acids, is a useful tool in characterizing the degree of membrane damage [47]. As shown in Figure 5A–C, the MDA level in the negative control group was significantly higher than that of the normal control cells, indicating that H_2_O_2_ treatment caused oxidative damage to HFL1 cells. By contrast, the MDA level decreased by 25.51%, 20.41%, and 23.67% after pretreatment with 200 μg/mL GDP2, GDP3, and GDP4, respectively, suggesting that GDPs alleviated the degree of membrane damage induced by H_2_O_2_ in the HFL1 cells.

Enzymatic antioxidant defense is a vital mechanism against ROS-induced oxidative stress in living organisms. Superoxide dismutase (SOD), the first line of defense system against ROS, can catalyze the conversion from O_2_^•^^−^ into H_2_O_2_, which can subsequently be eliminated by peroxidases such as catalase (CAT) and glutathione peroxidase (GSH-Px) while CAT plays a predominant role in decomposing H_2_O_2_ into harmless molecules [3]. The increase in antioxidant enzyme activity can reduce the oxidative stress of living cells [48]. In the present study, the activities of antioxidant enzymes (SOD, CAT, and GSH-Px) were measured to evaluating the protective effects of GDPs against H_2_O_2_-induced oxidative damage. As shown in Figure 5, SOD, CAT, and GSH-Px activities decreased by more than 50% after exposure to 200 μM H_2_O_2_ compared with those in the normal control group. However, GDPs significantly increased the antioxidant enzyme activities in the HFL1 cells under oxidative stress. SOD activity increased by 141.09%, 123.87%, and 62.79% after pretreatment with 200 μg/mL GDP2, GDP3, and GDP4, respectively, compared with that in the negative control cells (Figure 5A–C). Likewise, CAT activities in GDP2, GDP3, and GDP4 groups improved by 88.42%, 57.84%, and 52.13%, respectively, and GSH-Px activity increased by 36.66%, 47.00%, and 42.98%, respectively (Figure 5A–C). Similarly, polysaccharides from *S. fusiforme* can protect HaCaT human keratinocytes from oxidative stress by stimulating SOD and GSH-PX enzyme activities [41]. Moreover, GDP2 had the highest antioxidant activity. This result was consistent with the results obtained by determining cell viability and ROS level in the HFL1 cells (Figure 3 and Figure 4). Overall, all these results showed that GDPs can protect HFL1 cells from H_2_O_2_-induced oxidative damage by increasing the activities of antioxidant enzymes and scavenging free radicals. It is evidenced that antioxidant polysaccharides from red algae exert a protective role against oxidative stress primarily through eliminating ROS and activating the antioxidant system such as antioxidant enzymes [4], which is in line with our present results. However, the effects of GDP on oxidative stress-related signaling pathways at cellular and animal levels need to be further investigated.

### 2.9. GDP Inhibited Mitochondrial Membrane Potential (MMP) Decline in HFL1 Cells

**Figure 6:**
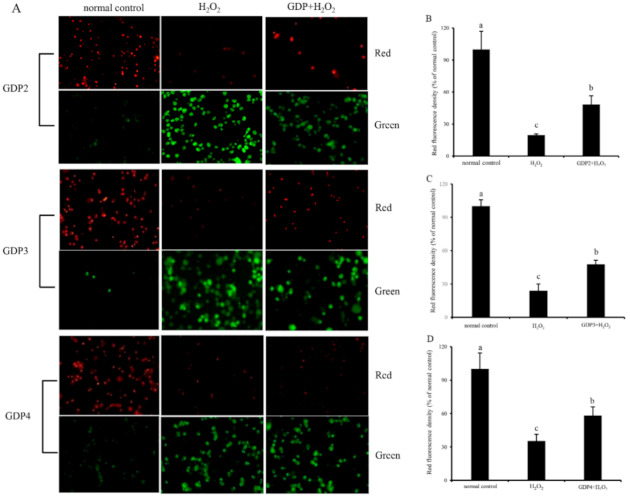
Effects of GDPs on the mitochondrial transmembrane potential (MMP) of HFL1 cells. JC-1 fluorescence detection (**A**); red fluorescence intensity values in different GDP treatment groups: GDP2 (**B**), GDP3 (**C**), and GDP4 (**D**). Vertical bars represent means ± SD (n = 4), and different letters indicate significant difference (*p* < 0.05).

Mitochondria play a critical role in cell survival [49]. H_2_O_2_-induced oxidative damage can lead to mitochondrial damage and dysfunction, affecting the regulation of mitochondrial redox balance [50]. As a biomarker of mitochondrial function, MMP is involved in many key events, such as oxidative stress and apoptosis [51]. Hence, MMP was determined for the evaluation of the protective effect of GDPs on the mitochondrial function of HFL1 cells against H_2_O_2_-induced oxidative damage. JC-1 can emit red fluorescence in the form of dimer in the mitochondrial matrix at a high membrane potential and emit green fluorescence as a monomer at a low membrane potential, and the red fluorescence intensity of JC-1 is commonly used to represent MMP level [52]. As shown in Figure 6, HFL1 cells treated with 200 μM H_2_O_2_ showed decreased red fluorescence intensity and enhanced green fluorescence intensity, indicating that H_2_O_2_-induced oxidative damage decreased MMP level and increased membrane permeability [52]. The red fluorescence intensities of the HFL1 cells pretreated with 200 μg/mL GDPs significantly increased compared with those of the negative control groups. The fluorescence intensities of GDP2-, GDP3-, and GDP4-treated cells increased by 145.43%, 99.04%, and 65.47%, respectively, indicating that GDPs pretreatment can inhibit a reduction in MMP levels and stabilize mitochondrial function.

## 3. Materials and Methods

### 3.1. Materials and Reagents

*G. lemaneiformis* was collected from the coast of Xiapu, Fujian, China (26°42′ N, 119°59′ E). Pectinase (500,000 U/g), glucoamylase (100,000 U/g), cellulase (50,000 U/g), xylanase (6,000,000 U/g), β-dextranase (50,000 U/g), and standard samples were purchased from Shanghai Yuanye Biotechnology Co, Ltd. (Shanghai, China). DPPH was purchased from McLean Biochemical Technology Co., Ltd., (Shanghai, China). All other chemicals were of analytical grade. The reagents and kits used in the cell experiment were purchased from Solarbio Science & Technology Co., Ltd. (Beijing, China). HFL1 cells and complete culture medium were obtained from iCell Bioscience Inc. (Shanghai, China).

### 3.2. GLP Extraction

*G. lemaneiformis* was washed thoroughly with water, dried at 50 °C for 48 h, and then ground into powder. The dried algal powder was soaked in distilled water in a ratio of 1:5 (*w*/*v*) for 24 h at room temperature for the removal of water-soluble proteins. Then, the powder was immersed in 95% ethanol in a ratio of 1:5 (*w*/*v*) for 24 h twice for the removal of pigments and lipids. Pretreated *G. lemaneiformis* powder was obtained by separating the residues through filtration and dried at 60 °C for 36 h.

Exactly 20 g of powdered algae was extracted twice with 1.0 L of hot water at 90 °C for 2 h. The extraction solutions were combined and concentrated to approximately one-fourth of their original volumes with a vacuum rotary evaporator at 55 °C and then precipitated with 95% ethanol with a volume that was four times the volumes of the original solutions at 4 °C overnight. After centrifugation (4000 rpm, 15 min), the precipitate was collected, washed with 95% ethanol two or three times, dissolved in distilled water, and vacuum freeze-dried to produce GLP.

### 3.3. Enzymatic Hydrolysis of GLP

GLP was hydrolyzed with different enzymes at optimum temperatures and pH (pectinase, 50 °C, pH 3.0; glucoamylase, 60 °C, pH 4.6; cellulose, 50 °C, pH 5.0; xylanase, 55 °C, pH 5.0; β-dextranase, 50 °C, pH 5.0) for 120 min at an enzyme dosage of 300,000 U/g. Enzymatic reactions were stopped by boiling the mixtures at 100 °C for 10 min for the inactivation of enzymes. The hydrolysates were then centrifuged, and each supernatant was dialyzed with 5000 Da of dialysis membranes at 4 °C for 24 h. Then degraded products produced by pectinase, glucoamylase, cellulose, xylanase, and β-dextranase were obtained after freeze-drying, and their antioxidant activities were determined through in vitro chemical assays for the screening of the optimal enzyme for GLP degradation.

### 3.4. Physicochemical Properties Analysis of Polysaccharides

The total sugar and protein content of the GLP were analyzed with the anthrone-sulfuric acid colorimetry method and Bradford’s method, respectively [53]. The contents of sulfate group and uronic acid in GLP and degradation products were determined with the barium sulfate turbidimetry method described by Lloyd et al. (1961) [54] and m-hydroxybiphenyl colorimetry method described by Blumenkrantz and Asboe-Hansen (1973) [55] with minor modification. The molecular weights of polysaccharides were analyzed through high-performance gel permeation chromatography [56]. Samples containing dextran with different molecular weights (1152, 11,600, 23,800, 48,600, 80,900, 148,000, 273,000, and 409,800 Da) were used as the standard samples for determining the purity and relative molecular weights of GLP and degradation products. The monosaccharide compositions of GLP and degradation products were analyzed using high-performance anion-exchange chromatography [57].

The chemical structures of GLP and degradation products were characterized using FT-IR spectra [58]. The GLP and degradation products were ground, and each sample (approximately 1 mg) was mixed with 100 mg KBr and pressed into a translucent wafer under an anhydrous condition. Then, the mixture was analyzed and scanned through FT-IR spectroscopy (FT-IR-4700, Jasco, Japan) at a wavelength range of 400–4000 cm^−1^.

### 3.5. Single-Factor and Orthogonal Optimization Experiments

On the basis of the above experiments, the effects of hydrolysis time (60, 90, 120, or 150 min), hydrolysis temperature (30, 40, 50, or 60 °C), hydrolysis pH (3.8, 4.4, 5.0, or 5.6), and enzyme dosage (200,000, 250,000, 300,000, or 350,000 U/g) on the antioxidant activities of the degradation products were studied through single-factor experiments for GLP degradation.

According to the results of single-factor experiments, three factors, including temperature (30, 40, and 50 °C), pH (4.4, 5.0, and 5.6), and enzyme dosage (250,000, 300,000, and 350,000 U/g) were used in designing an L_9_(3^3^) orthogonal experiment for the optimization of the degradation process. The factors and levels of the orthogonal design were shown in Table S2.

### 3.6. In Vitro Chemical Assays of Antioxidant Activity

#### 3.6.1. Hydroxyl Radical Scavenging Activity

The samples were dissolved in distilled water for the preparation of 0.2, 0.4, 0.6, 0.8, and 1.0 mg/mL sample solutions. A total of 0.5 mL of each sample solution was mixed with 0.5 mL of 9 mM FeSO_4_, 0.5 mL of 8.8 mM H_2_O_2_, and 0.5 mL of 9 mM salicylic acid ethanol solution. The reaction solution was incubated at 37 °C for 30 min and then measured at 510 nm. Distilled water was used as the control. The scavenging capacity of the hydroxyl radical was calculated using the following equation [59]:

Scavenging percentage (%) = (1 − *A*_1_/*A*_0_) × 100% (1)

where *A*_1_ is the absorbance of the sample and *A*_0_ is the absorbance of the control (distilled water).

#### 3.6.2. DPPH Radical Scavenging Activity

DPPH radical scavenging activity determination was assayed through the method as described by You et al. (2011) [60] with slight modification. Sample solutions of 0.2, 0.4, 0.6, 0.8, and 1.0 mg/mL were prepared with distilled water. Then, 1.0 mL of sample solution was mixed with 1.0 mL of 0.2 mM DPPH dissolved in anhydrous ethanol and vortexed. The mixture was incubated at 37 °C for 30 min in the dark, and absorbance was read at 517 nm. Distilled water was used as the control. The scavenging capacity of DPPH radical was calculated using the following equation:

Scavenging percentage (%) = [1 − (*A_i_* − *A_j_*)/*A*_0_] × 100% (2)

where *A_i_* is the absorbance of the sample mixed with the DPPH solution, *A_j_* is the absorbance of the sample mixed with anhydrous ethanol, and *A*_0_ is the absorbance of distilled water mixed with the DPPH solution.

#### 3.6.3. Reduction Capacity

The reducibility of the GLP and degradation products were determined by the potassium ferricyanide method described by Wang et al. (2019) [30] with slight modification. A mixture containing 0.5 mL of sample solution, 0.5 mL of PBS (2 M, pH 6.6), and 0.5 mL of 1% potassium ferricyanide were prepared and incubated at 50 °C for 20 min. The reaction was stopped by adding 0.5 mL of 10% TCA after rapid cooling, and then the mixture was centrifuged at 3000 rpm for 10 min. A total of 1.0 mL of the supernatant was mixed with 1.0 mL of distilled water and 0.2 mL of 0.1% FeSO_4_ and left for 10 min, and absorbance was measured at 700 nm.

#### 3.6.4. Total Antioxidant Capacity

For total antioxidant capacity determination, 0.1 mL sample solution was mixed with 3 mL of FRAP working solution (FRAP working solution was prepared with 0.3 M CH_3_COONa buffer solution, 20 mM FeCl_3_, and 10 mM TPTZ in a ratio of 10:1:1) and 0.3 mL of distilled water. Absorbance was measured at 593 nm after incubation at 37 °C for 20 min. An increase in the absorbance of the sample indicated the enhancement of the total antioxidant capacity [61].

### 3.7. In Vitro Biological Assays of Antioxidant Activity

#### 3.7.1. Cell Culture and Cell Viability Assay

The effects of GDP2, GDP3, and GDP4 on the antioxidant activity of the HFL1 cells treated with H_2_O_2_ were investigated. HFL1 cells were cultured in Dulbecco’s modification of Eagle’s medium high-glucose medium (containing 10% fetal bovine serum, 100 UI/mL penicillin, and 100 μg/mL streptomycin) at an atmosphere of 90% relative humidity, 5% CO_2_, and 37 °C. Cell viability was determined with the MTT method. HFL1 cells in the logarithmic growth phase were precultured in 96-well plates (2 × 10^3^ cells/well) for 24 h. Different concentrations of the samples (0, 6.25, 12.5, 25, 50, 100, 200, and 400 μg/mL) were added and cultured for 12 h, then treated with 200 μM H_2_O_2_ for another 6 h. After exposure to H_2_O_2_, 40 μL of MTT solution (5 mg/mL) was added to each well and incubated at 37 °C for 4 h. Approximately 150 μL of DMSO was added to each well for the dissolution of the crystal, and absorbance was measured at 570 nm for the calculation of relative cell viability. Each treatment was repeated in four wells. The cell viability of the normal control group was 100%.

#### 3.7.2. Measurement of Intracellular ROS Generation

ROS content was detected through fluorescence microscopy with fluorescent probe 2′,7′-dihydrofluorescein-diacetate (DCFH-DA) [45]. In detail, HFL1 cells were seeded in 96-well black plates at a density of 2 × 10^3^ cells/well and cultured for 24 h before treatment. The cells were treated with 200 μg/mL samples and 200 μM H_2_O_2_ and then with 10 μM DCFH-DA for 30 min at 37 °C in the dark. The fluorescence intensity of DCF was determined at an excitation wavelength of 485 nm and an emission wavelength of 535 nm. Pictures were obtained using a fluorescence microscope (80I, Nikon, Japan). Relative ROS production was expressed as the percentage of DCF fluorescence of the control.

#### 3.7.3. Cellular Antioxidant Activity Measurement

HFL1 cells were seeded in six-well plates at a density of 1 × 10^6^ cells/well and treated with different concentrations of GDP2, GDP3, or GDP4 for 12 h and then with 200 μM H_2_O_2_ for 6 h. The cells were collected and treated with cell lysis buffer. The lysed cells were used in measuring MDA content and SOD, CAT, and GSH-Px activities according to the manufacturer’s instructions of the kits used.

#### 3.7.4. Determination of MMP

MMP was measured using a JC-1 kit [52]. HFL1 cells were seeded in 96-well black plates at a density of 2 × 10^3^ cells/well and incubated for 24 h before treatment. After GDP and H_2_O_2_ treatments, the cells were cultured in a complete medium containing JC-1 (10 μg/mL) at 37 °C for 30 min in the dark. The fluorescence intensities of the cells were measured at excitation wavelengths of 514 and 585 nm and emission wavelengths of 529 and 590 nm.

### 3.8. Statistical Analysis

Data were expressed as means ± standard deviation (SD, n ≥ 3). One-way analysis of variance (ANOVA) was used in evaluating differences between different groups with SPSS 22.0. The confidence level was set at a *p*-value of < 0.05.

## 4. Conclusions

In the present study, five low-molecular-weight polysaccharides (GPP, GGP, GCP, GXP, and GDP) with different Mw and monosaccharide compositions were prepared from *G. lemaneiformis* by the enzymatic degradation of GLP for the first time. The degraded products possessed higher antioxidant activity than natural GLP, and GDP was the most effective, which may be ascribed to the comprehensive effects of the variations of its total sugar content, molecular weight, and monosaccharide composition. After the optimization of the degradation conditions of GDP, the in vitro antioxidant activity of GDP was significantly enhanced in a dose-dependent manner, and four polysaccharide fractions (GDP1, GDP2, GDP3, and GDP4) with excellent antioxidant activities were obtained. Furthermore, GDPs (GDP2, GDP3, and GDP4) significantly enhanced cell viability and antioxidant enzyme activity, reduced ROS and MDA levels, and inhibited the reduction in MMP level in HFL1 cells. In line with its better DPPH free radical scavenging ability, GDP2 possessed the best cytoprotection effect on oxidative damaged HFL1 cells. GDP not only has suitable in vitro free radical scavenging capacity but also can protect HFL1 cells from oxidative damage by activating cellular antioxidant enzyme activity. Thus, enzyme degradation with β-dextranase can be a promising method to prepare GLP with higher antioxidant activity, and GDP might be a suitable source of natural antioxidants.

## Figures and Tables

**Table 1:** Physicochemical properties of the samples.

Samples	Total Sugar/%	Sulfate/%	Uronic Acid/%	Mw/kDa
GLP	69.33 ± 0.61	18.71 ± 1.31	5.55 ± 0.20	106
GPP	74.29 ± 1.48 *	13.91 ± 0.89 **	6.40 ± 0.08 **	66
GGP	78.21 ± 4.21 **	18.91 ± 1.12	5.32 ± 0.38	93
GCP	74.29 ± 2.86 *	4.56 ± 0.48 **	4.13 ± 0.09 **	89
GXP	72.68 ± 2.35	7.33 ± 0.10 **	6.01 ± 0.11 *	95
GDP	79.13 ± 0.91 **	10.11 ± 0.56 **	5.46 ± 0.14	81

* *p* < 0.05 and ** *p* < 0.01.

**Table 2:** Monosaccharide composition of the samples.

Monosaccharide Composition (mol %)	GLP	GPP	GGP	GCP	GXP	GDP
Fucose (Fuc)	-	-	-	-	0.2	-
Rhamnose (Rha)	-	1.4	1.2	-	-	0.7
Arabinose (Ara)	-	0.2	0.5	-	-	0.1
Galactose (Gal)	89.7	58.8	68.0	20.0	70.9	41.9
Glucose (Glu)	2.3	33	13.8	74.5	23.4	42.1
Xylose (Xyl)	1.2	1.5	1.0	0.1	0.6	0.8
Mannose (Man)	-	-	10.9	4.2	0.8	11.1
Galacturonic acid (GalA)	3.4	2.2	2.6	0.4	1.6	1.3
Glucuronic acid (GluA)	3.4	2.1	1.3	0.6	1.4	0.6
Mannuronic acid (ManA)	-	-	-	-	0.7	0.5

**Table 3:** Design and corresponding results of orthogonal experiment L_9_(3^3^) based on the antioxidant capacities of the GLP hydrolysates.

Number		A (Temperature/°C)	B (pH)	C (Enzyme Dosage/U·g^−1^)	Hydroxyl Radical Scavenging Rate/%	DPPH Radical Scavenging Rate/%	Reducing Capacity	Total Antioxidant Capacity	Comprehensive Score
1		1(30)	1(4.4)	1(250,000)	48.33 ± 0.26	29.60 ± 0.38	0.096 ± 0.002	0.322 ± 0.002	19.59
2		1(30)	2(5.0)	2(300,000)	44.22 ± 0.85	28.42 ± 0.14	0.108 ± 0.002	0.329 ± 0.001	18.27
3		1(30)	3(5.6)	3(350,000)	35.65 ± 0.64	28.79 ± 0.38	0.104 ± 0.001	0.320 ± 0.001	16.22
4		2(40)	1(4.4)	2(300,000)	43.39 ± 1.43	19.65 ± 1.08	0.109 ± 0.004	0.324 ± 0.001	15.87
5		2(40)	2(5.0)	3(350,000)	40.27 ± 2.71	20.74 ± 0.05	0.118 ± 0.004	0.326 ± 0.001	15.36
6		2(40)	3(5.6)	1(250,000)	38.92 ± 0.17	22.45 ± 0.35	0.117 ± 0.001	0.324 ± 0.001	15.45
7		3(50)	1(4.4)	3(350,000)	37.12 ± 1.13	22.23 ± 0.11	0.124 ± 0.003	0.322 ± 0.001	14.95
8		3(50)	2(5.0)	1(250,000)	41.94 ± 0.16	17.69 ± 0.53	0.117 ± 0.003	0.322 ± 0.001	15.02
9		3(50)	3(5.6)	2(300,000)	39.30 ± 0.61	15.92 ± 0.14	0.124 ± 0.006	0.322 ± 0.001	13.92
Hydroxyl radical scavenging rate/%	K_1_	42.73	42.95	43.06					
	K_2_	40.86	42.14	42.30					
	K_3_	39.45	37.96	37.68					
	R	3.28	4.99	5.38					
DPPH radical scavenging rate/%	K_1_	28.94	23.83	23.25					
	K_2_	20.95	22.28	21.33					
	K_3_	18.61	22.39	23.92					
	R	10.32	1.54	2.59					
Reducing capacity	K_1_	0.103	0.110	0.110					
	K_2_	0.115	0.114	0.114					
	K_3_	0.122	0.115	0.115					
	R	0.019	0.005	0.005					
Total antioxidant capacity	K_1_	0.324	0.323	0.323					
	K_2_	0.325	0.326	0.325					
	K_3_	0.322	0.322	0.323					
	R	0.003	0.004	0.002					
Comprehensive score	K_1_	18.03	16.80	16.69					
	K_2_	15.56	16.22	16.02					
	K_3_	14.63	15.20	15.51					
	R	3.40	1.61	1.18					

**Table 4:** Results of verification experiment for the antioxidant activities of GDPs obtained at optimal conditions. Data represent means ± SD (n = 3), and different letters in the same column indicate significant difference (*p* < 0.05).

Samples	Scheme	Hydroxyl Radical Scavenging Rate/%	DPPH Radical Scavenging Rate/%	Reducing Capacity	Total Antioxidant Capacity
GDP1	A_1_B_1_C_1_	52.20 ± 0.82b	29.73 ± 0.71b	0.117 ± 0.002c	0.325 ± 0.001d
GDP2	A_1_B_1_C_3_	39.92 ± 0.63d	32.68 ± 0.75a	0.125 ± 0.004b	0.334 ± 0.002c
GDP3	A_2_B_2_C_2_	61.99 ± 2.44a	24.28 ± 1.08d	0.129 ± 0.002b	0.345 ± 0.002b
GDP4	A_3_B_3_C_3_	44.27 ± 0.16c	26.15 ± 0.23c	0.140 ± 0.001a	0.355 ± 0.002a

## Data Availability

The data are contained within the article or Supplementary Materials and can be available from the corresponding author upon reasonable request.

## Supplementary Materials

The following are available online at https://www.mdpi.com/article/10.3390/md19050270/s1, Figure S1: FTIR spectrum of GLP and its different enzymatic hydrolysis products: GLP(A), GPP(B), GGP(C), GCP(D), GXP(E), GDP(F), Figure S2: Effects of enzymolysis time on scavenging ability of hydroxyl radical (A), scavenging ability of DPPH radical (B), reducing capacity (C) and total antioxidant capacity (D) of GDP. Vertical bars represent means ± SD (n = 3). Different uppercase letters among different concentration of the same group indicate significant difference (*p* < 0.05), and different lowercase letters among different groups of the same concentration indicate significant difference (*p* < 0.05), Figure S3: Effects of enzymolysis temperature on scavenging ability of hydroxyl radical (A), scavenging ability of DPPH radical (B), reducing capacity (C) and total antioxidant capacity (D) of GDP. Vertical bars represent means ± SD (n = 3). Different uppercase letters among different concentration of the same group indicate significant difference (*p* < 0.05), and different lowercase letters among different groups of the same concentration indicate significant difference (*p* < 0.05), Figure S4: Effects of enzymolysis pH on scavenging ability of hydroxyl radical (A), scavenging ability of DPPH radical (B), reducing capacity (C) and total antioxidant capacity (D) of GDP. Vertical bars represent means ± SD (n = 3). Different uppercase letters among different concentration of the same group indicate significant difference (*p* < 0.05), and different lowercase letters among different groups of the same concentration indicate significant difference (*p* < 0.05), Figure S5: Effects of enzyme dosage on scavenging ability of hydroxyl radical (A), scavenging ability of DPPH radical (B), reducing capacity (C) and total antioxidant capacity (D) of GDP. Vertical bars represent means ± SD (n = 3). Different uppercase letters among different concentration of the same group indicate significant difference (*p* < 0.05), and different lowercase letters among different groups of the same concentration indicate significant difference (*p* < 0.05), Table S1: Optimal conditions obtained from the analysis of orthogonal experiment L_9_(3^3^) based on different evaluation indexes, Table S2: The factors and levels of orthogonal design.
