# Supplementary material for: Enzymatic Degradation of Gracilariopsis lemaneiformis Polysaccharide and the Antioxidant Activity of Its Degradation Products
Source: Mar Drugs. 2021 May 12;19(5):270. doi: 10.3390/md19050270 (PMC8150296; doi:10.3390/md19050270)
Supplement: Supplementary file 1 [file marinedrugs-19-00270-s001.zip › marinedrugs-1192573Supplementary File (1).pdf]

Supplementary materials

Table S1. Optimal conditions obtained from the analysis of orthogonal experiment $L_9(3^3)$ based on different evaluation indexes.

**Evaluation index**	**Range & Optimal Scheme**	**Factor A (Temperature)**	**Factor B (pH)**	**Factor C (Enzyme dosage)**
Hydroxyl radical scavenging rate/%	R	3.28	4.99	5.38
	Optimal Scheme	A_1	B_1	C_1
DPPH radical scavenging rate/%	R	10.32	1.54	2.59
	Optimal Scheme	A_1	B_1	C_3
Reducing capacity	R	0.019	0.005	0.005
	Optimal Scheme	A_3	B_3	C_3
Total antioxidant capacity	R	0.003	0.004	0.002
	Optimal Scheme	A_2	B_2	C_2
Comprehensive score	R	3.40	1.61	1.18
	Optimal Scheme	A_1	B_1	C_1

Table S2. The factors and levels of orthogonal design.

Levels	Factors		
	Temperature (℃)	pH	Enzyme dosage （U/g）
1	30	4.4	250,000
2	40	5.0	300,000
3	50	5.6	350,000

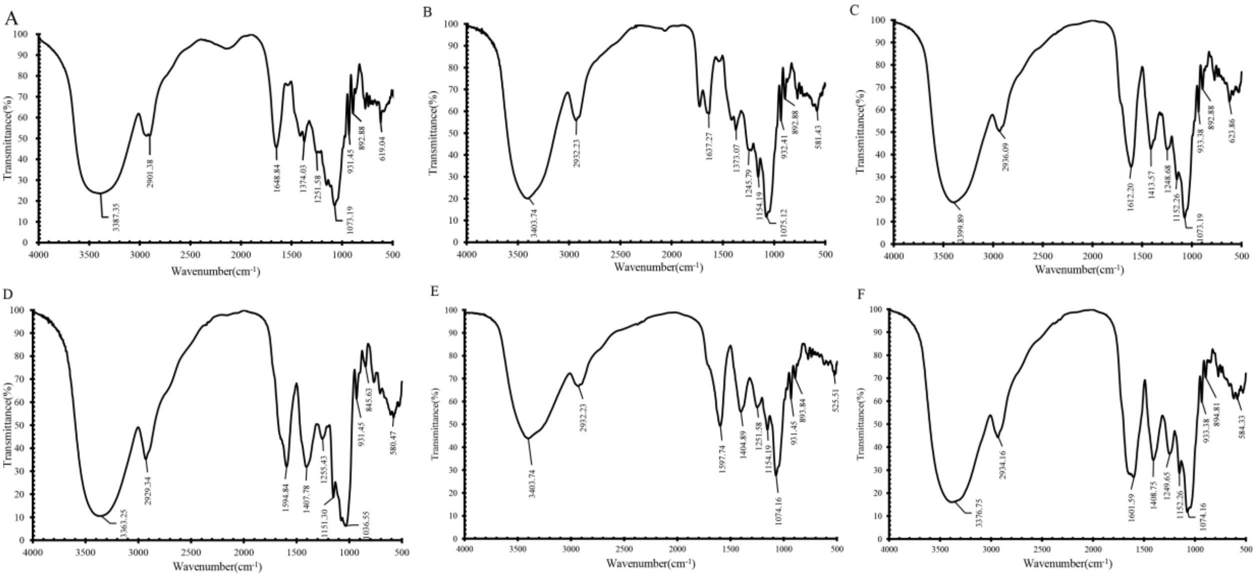

Figure S1 FTIR spectrum of GLP and its different enzymatic hydrolysis products: GLP(A), GPP(B), GGP(C), GCP(D), GXP(E), GDP(F).

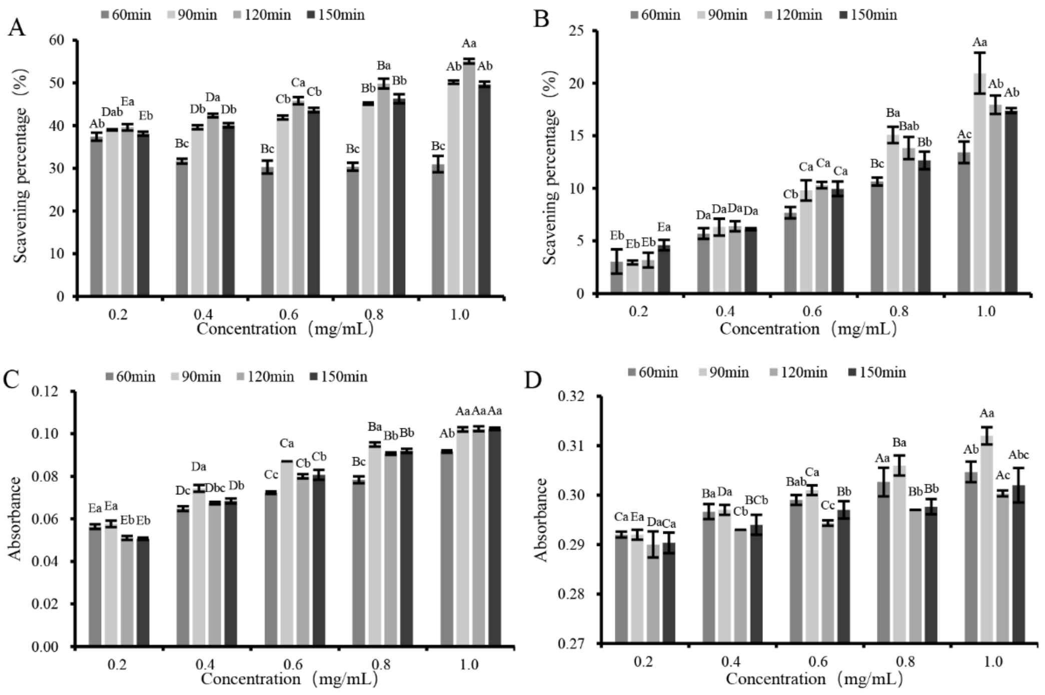

Figure S2 Effects of enzymolysis time on scavenging ability of hydroxyl radical (A), scavenging ability of DPPH radical (B), reducing capacity (C) and total antioxidant capacity (D) of GDP. Vertical bars represent means ± SD (n = 3). Different uppercase letters among different concentration of the same group indicate significant difference ($p < 0.05$), and different lowercase letters among different groups of the same concentration indicate significant difference ($p < 0.05$).

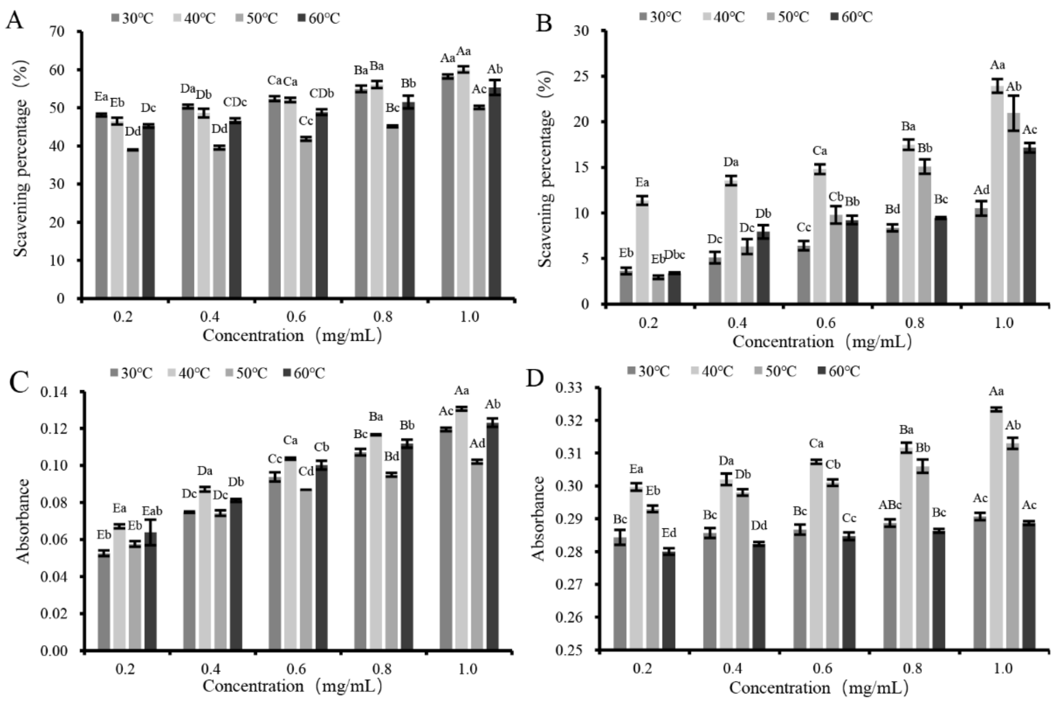

Figure S3 Effects of enzymolysis temperature on scavenging ability of hydroxyl radical (A), scavenging ability of DPPH radical (B), reducing capacity (C) and total antioxidant capacity (D) of GDP. Vertical bars represent means ± SD (n = 3). Different uppercase letters among different concentration of the same group indicate significant difference ($p < 0.05$), and different lowercase letters among different groups of the same concentration indicate significant difference ($p < 0.05$).

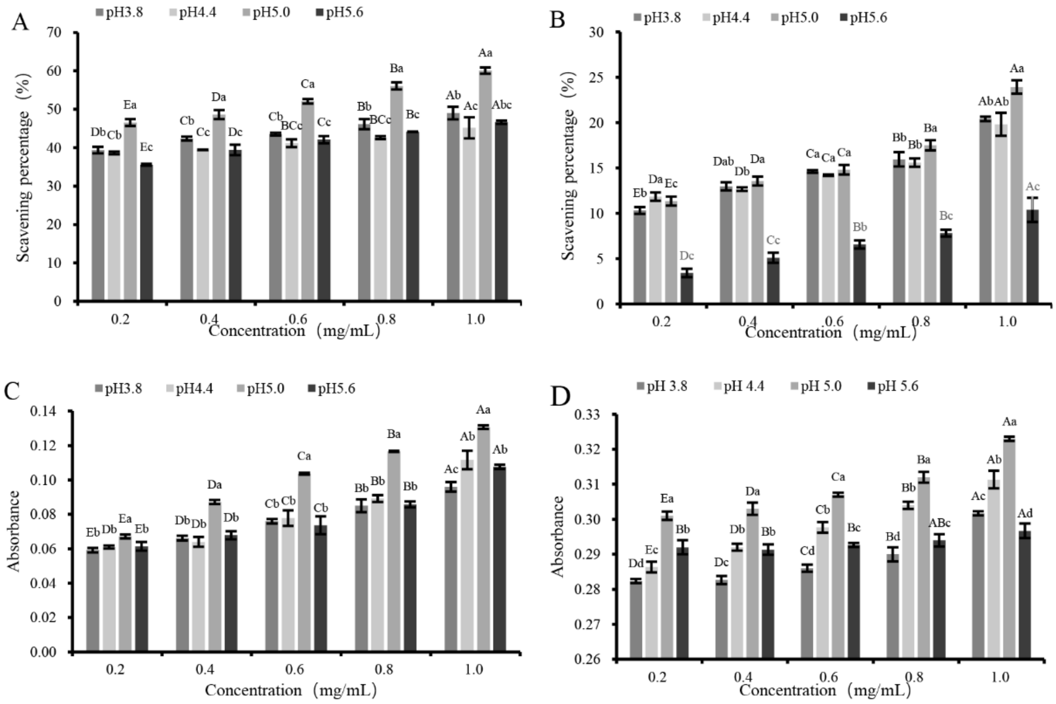

Figure S4 Effects of enzymolysis pH on scavenging ability of hydroxyl radical (A), scavenging ability of DPPH radical (B), reducing capacity (C) and total antioxidant capacity (D) of GDP. Vertical bars represent means ± SD (n = 3). Different uppercase letters among different concentration of the same group indicate significant difference ($p < 0.05$), and different lowercase letters among different groups of the same concentration indicate significant difference ($p < 0.05$).

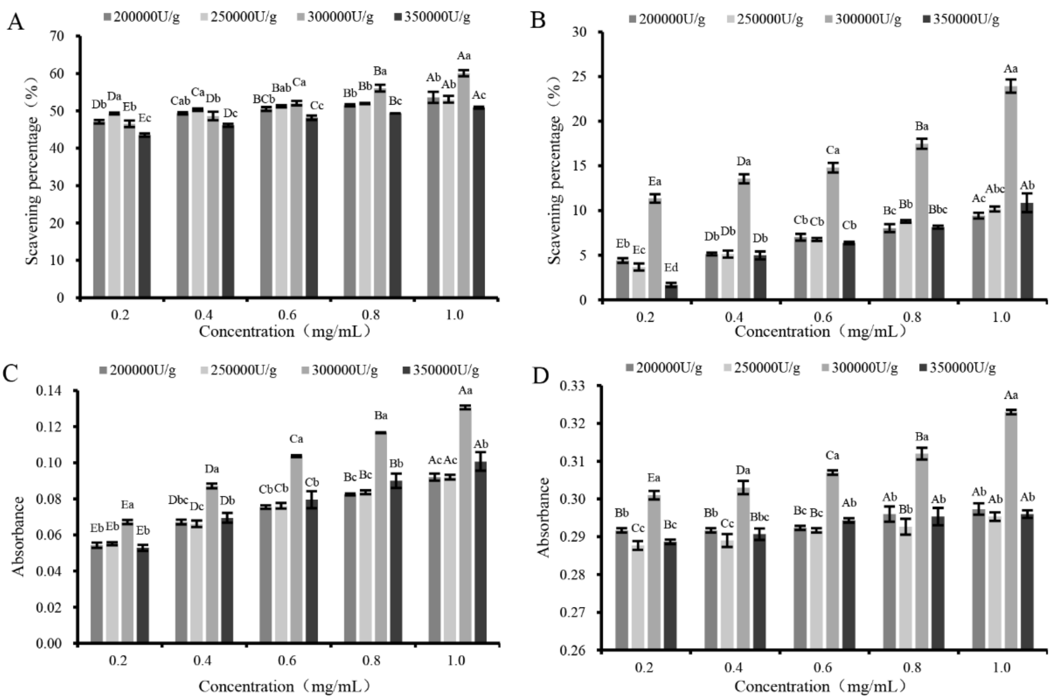

Figure S5 Effects of enzyme dosage on scavenging ability of hydroxyl radical (A), scavenging ability of DPPH radical (B), reducing capacity (C) and total antioxidant capacity (D) of GDP. Vertical bars represent means ± SD (n = 3). Different uppercase letters among different concentration of the same group indicate significant difference ($p < 0.05$), and different lowercase letters among different groups of the same concentration indicate significant difference ($p < 0.05$).
